# Evaluating Pharmacokinetic and Pharmacodynamic Interactions with Computational Models in Supporting Cumulative Risk Assessment

**DOI:** 10.3390/ijerph8051613

**Published:** 2011-05-19

**Authors:** Yu-Mei Tan, Harvey Clewell, Jerry Campbell, Melvin Andersen

**Affiliations:** 1 National Exposure Research Laboratory, Office of Research and Development, U.S. Environmental Protection Agency, 109 T.W. Alexander Drive, Research Triangle Park, NC 27711, USA; 2 Center for Human Health Assessment, The Hamner Institutes for Health Sciences, 6 Davis Drive, Research Triangle Park, NC 27709, USA; E-Mails: hclewell@thehamner.org (H.C.); jcampbell@thehamner.org (J.C.); mandersen@thehamner.org (M.A.)

**Keywords:** pharmacokinetics, pharmacodynamics, mixture interactions, physiologically based pharmacokinetic/pharmacodynamic model

## Abstract

Simultaneous or sequential exposure to multiple chemicals may cause interactions in the pharmacokinetics (PK) and/or pharmacodynamics (PD) of the individual chemicals. Such interactions can cause modification of the internal or target dose/response of one chemical in the mixture by other chemical(s), resulting in a change in the toxicity from that predicted from the summation of the effects of the single chemicals using dose additivity. In such cases, conducting quantitative cumulative risk assessment for chemicals present as a mixture is difficult. The uncertainties that arise from PK interactions can be addressed by developing physiologically based pharmacokinetic (PBPK) models to describe the disposition of chemical mixtures. Further, PK models can be developed to describe mechanisms of action and tissue responses. In this article, PBPK/PD modeling efforts conducted to investigate chemical interactions at the PK and PD levels are reviewed to demonstrate the use of this predictive modeling framework in assessing health risks associated with exposures to complex chemical mixtures.

## 1. Introduction

**Figure 1.**
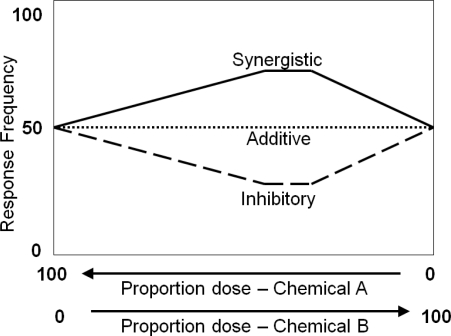
The traditional empirical approach for examining dose additivity between chemicals A and B. Additive: Same response from the mixture is observed from the sum of A and B. Synergism: A greater response from the mixture is observed than expected from the sum of A and B. Inhibitory: A lower response from the mixture is observed than expected from the sum of A and B.

A challenge for estimating cumulative risks across multiple chemicals is determining whether the responses generated from exposures to multiple chemicals simultaneously are different from estimates based on the addition of individual responses. The hypothesis of dose additivity among chemicals in a mixture is typically evaluated through empiricism. If a greater response from the mixture is observed than expected from the sum of the individual chemicals, the mixture response is described as synergism or potentiation (Figure 1). If a lower mixture response is observed than expected, the mixture response is described as antagonism or inhibition (Figure 1). To better distinguish additivity and non-additivity, many efforts have concentrated on improving the analytical capability to more accurately measure responses or on developing statistical methods to properly test the null hypothesis of dose additivity [1]. Even with the most advanced analytical and statistical tools, however, determining the cumulative risk from exposure to environmental chemicals is still a difficult challenge.

In the absence of toxicity data on the mixture of concern, data on similar/related mixtures or data on individual chemicals in the mixture may be used for assessing cumulative risk from mixture exposures. Examples of methods for conducting cumulative risk assessment include the Hazard Index (HI) approach, weight-of-evidence modification of the HI approach, the toxicity equivalency factor approach, and the relative potency factor approach [2–4]. These methods, however, lack a mechanistic basis and thus are of limited utility for high-to-low dose or animal-to-human extrapolations. For example, a study on a quaternary mixture of trihalomethanes (THMs) showed that the rat venous blood concentrations of each THM following the mixture exposure were significantly higher compared to blood concentrations observed after exposures to single chemicals [5]. This finding is consistent with the occurrence of mutual inhibition of hepatic metabolism from THMs. Without considering this kinetic information (*i.e.*, competitive inhibition), one may not recognize that this non-additive result is unlikely to occur in humans since the threshold for significant metabolic inhibition is not reached at environmental concentrations.

**Figure 2.**
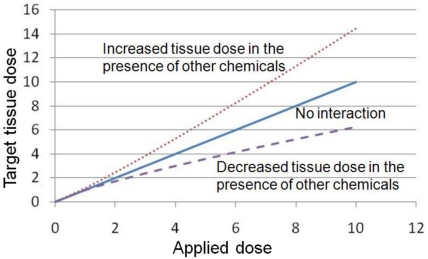
The impact of pharmacokinetic (PK) interactions on target tissue dose. The term “PK interaction” refers to the case in which one unit of applied dose to chemical X in the presence of other chemicals leads to less (examples in Section 3.1) or more (examples in Section 3.2) than one unit of target tissue dose compared to exposure to chemical X by itself.

**Figure 3.**
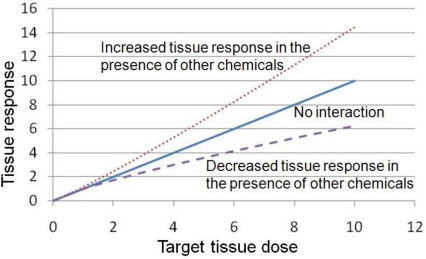
The impact of pharmacodynamic (PD) interactions on tissue response. The term “PD interaction” refers to the case in which one unit of target tissue dose to chemical X in the presence of other chemicals leads to less (examples in Section 4.1) or more (examples in Section 4.2) than one unit of tissue response compared to exposure to chemical X by itself.

When assessing the toxicity and the resulting risk of chemical mixtures, a more mechanistic approach should be taken to characterize the interactions among individual chemicals in a mixture. In this article, two types of interactions will be discussed: Pharmacokinetic (PK) and pharmacodynamic (PD) interactions [6]. Interactions related to cell signaling networks that can affect a common physiological process are also important when assessing cumulative risk, but are not the focus of this article. Simply put, PK depicts the process of chemicals being absorbed, distributed to, metabolized within, and eliminated (ADME) from various organs and tissues. The term ‘PK interaction’ refers to the case in which one unit of applied dose to chemical “X” in the presence of other chemicals leads to more or less than one unit of target tissue dose compared to exposure to chemical “X” by itself (Figure 2). Most interactions studied to date are PK interactions. On the other hand, PD describes how chemicals bring about tissue responses. The term “PD interaction” refers to the case which one unit of target tissue dose brought about by chemical “X” in the presence of other chemicals leads to more or less than one unit of tissue response compared to response caused by chemical “X” by itself (Figure 3). With the proper identification of interactions occurring at various levels, a more integrated systems approach can then be applied to provide a better perspective on how both biology and biochemistry impact mixture toxicity and cumulative risk.

## 2. Computational Modeling of Chemical Interactions

The knowledge on PK or PD interactions can be integrated in a quantitative manner with a mechanistic model. A mechanistic model for chemical mixtures is composed of three major elements: (1) The interaction among individual chemicals in the mixture at the level of PK and PD; (2) Quantitative descriptions of both temporal (*i.e*., concurrent or sequential exposures) and dose relationships among individual chemicals; and (3) Each chemical’s mode of action. Mode of action is the sequence of events by which the active form of the chemical (parent or metabolites) interacts with the target tissue and leads to responses. A commonly used computational model that consists of all three elements described above is a physiologically based pharmacokinetic/pharmacodynamic (PBPK/PD) model.

PBPK modeling involves the development of mass-balance differential equations to describe the ADME processes of chemicals as a function of their physiochemical (e.g., tissue:blood partition coefficient), biochemical (e.g., metabolic rate constant), and physiological (e.g., tissue volume) characteristics. Typically, a PBPK model consists of a series of biologically relevant compartments, each receiving the chemical via the arterial blood and returning the free chemical via the venous blood. These compartments may represent a single tissue or a group of tissues with similar blood flow and solubility characteristics.

PBPK models of chemical mixtures involve the change of rates of ADME for one or more chemicals. The alteration of the absorption/excretion rate of a chemical as a result of the presence of other chemicals is often caused by interference with an active uptake/excretion process or by modulation of critical biological determinants of uptake (e.g., breathing rate) or excretion (e.g., glomerular filtration). The distribution rate can change when multiple chemicals compete for binding to the same macromolecules (e.g., hemoglobin, albumin, metallothionein) or proteins. The enhancement of metabolic rate (induction) can occur in the case when specific isoenzymes are induced by prior exposures to certain chemicals, leading to an increase in the enzyme capacity to metabolize other chemicals. The reduction of metabolic rate (inhibition) is a result of two or more chemicals competing for biotransformation mediated by the same enzyme. With environmental chemicals, the most common single mechanism of interaction investigated is inhibition or induction of hepatic cytochrome P450 by mixtures of chemicals [7,8]. Metabolism plays an important role in the toxicity of many chemicals, either by the detoxification of a directly toxic compound or by the formation of a metabolite that is more reactive than the parent compound [9].

Besides simulating the ADME processes, PBPK models also have the capability to simulate target tissue dose, which is the determinant of tissue reactions leading to toxicity [10]. Thus, a PBPK model can be connected to a PD model to simulate the time course of tissue response as a function of target tissue dose. The quantitative descriptions of PK and PD of individual chemicals in PBPK/PD models allows one to investigate the possibility and degree of interactions among chemicals in the PK and PD processes at different exposure scenarios. This modeling approach provides a means to evaluate mixture interactions and associated cumulative risk that are not readily available from observing the temporal and dose-response relationship of co-existing chemicals (*i.e.*, empiricism). In addition, modeling approach has the capability to answer questions for experiments that would be too costly and complex to perform.

Another advantage of analyzing mixture interactions with a PBPK model is that mixture exposure may result in altering one biomarker while other biomarkers of the same chemical remain unchanged. For example, Tardif and Charest-Tardif exposed rats for 4 h to 1,1,1-trichloroethane or *m*-xylene alone or as a mixture and found that the blood concentration of 1,1,1-trichloroethane was not affected whereas that of *m*-xylene was increased [11]. They also found that the excretion of 1,1,1-trichloro-ethane metabolites (trichloroethanol and trichloroacetic acid) during a period of 48 h following the onset of exposure was significantly reduced [11]. These results were successfully simulated using a PBPK model with a description of competitive inhibition in the liver [7]. This example highlights the importance of conducting quantitative analysis of PK and PD interactions before selecting appropriate biomarkers to study mixture interactions. In the following sections, examples will be provided to further illustrate mixture interactions at the PK and PD levels. In addition, these examples will demonstrate how computational models can be used to enhance our ability to evaluate cumulative risk following mixture exposures by incorporating the interaction mechanisms.

## 3. Examples of Pharmacokinetic Interactions among Mixtures

### 3.1. Decreased Tissue Dose in the Presence of Other Chemicals

#### 3.1.1. Trichloroethylene (TCE) in a Mixture

TCE is a common organic solvent used in industry worldwide; it is also a widespread environmental chemical. In animal studies, TCE has shown to induce various toxicological effects in kidneys, liver, and lungs that may be attributable to its metabolites [12]. Several studies were conducted to investigate the toxicological interactions between TCE, its metabolites, and other chemicals including solvents, haloacetates, and ethanol [13]. In this section, two of these studies are presented on the suppressive effects on TCE metabolism due to co-exposure to other chemicals. The first study is on the mixture of TCE, perchloroethylene (PERC) and 1,1,1-trichloroethane (methyl chloroform, MC) [14]; this mixture is listed on the U.S. Environmental Protection Agency (EPA) National Priorities List (NPL) among the most frequently detected ternary mixtures in air at the current and former hazardous waste sites [15]. As these three chemicals undergo concurrent P450-catalyzed oxidation in liver, potential PK interactions (and subsequent impact on toxicity) may occur [16,17]. To investigate the interaction, Dobrev and colleagues built three PBPK models, one for each chemical, that share an identical model structure [14]. Then, these three models were linked by implementing alternative inhibitory metabolism equations (competitive, uncompetitive, and non-competitive) in the liver compartments. Model simulations using each of the alternative equations were compared to chemical concentrations measured in the gas phase of the closed-chamber to determine which inhibition mechanism is most plausible. Dobrev and colleagues found that the competitive inhibition equation best described the pharmacokinetics of the ternary mixture: Co-exposure to PERC and MC result in a significant reduction of TCE metabolism at high exposure concentrations, but less so at low environmental exposure concentrations [14].

The second TCE study investigated the basis for decrease in 1,1-dichloroethylene (DCE) toxicity when rats were co-exposed to TCE [18]. 1,1-DCE is a potent hepatotoxicant that exerts acute toxicity when its reactive metabolites are formed faster than they can be detoxified by glutathione (GSH) [19]. Since both 1,1-DCE and TCE are metabolized by CYP2E1 in the liver, Andersen and colleagues built a PBPK model for the binary mixture with each chemical as an inhibitor of the other’s metabolism [18]. Similar to the study described above [14], multiple mechanisms of inhibitory interactions were examined by comparing model simulations with measured chemicals concentrations in gas chamber [18]. The best correspondence between predicted and observed time course behaviors was obtained when the inhibition was assumed to be competitive. In addition, the PBPK model with the competitive inhibition equation was able to predict the decreased 1,1-DCE hepatotoxicity (serum aspartate transaminase as a surrogate) due to decreased metabolism when co-exposed to TCE [18]. It is important to note that a PBPK model has the capability to analyze co-exposure effects on metabolism and toxicity due to other PK interactions. For example, a PBPK model that describes GSH depletion associated with metabolism can be used to evaluate the alteration of 1,1-DCE hepatotoxicity as a result of co-exposure to vinyl chloride, since both chemicals deplete GSH due to a formation of reactive metabolites [20].

#### 3.1.2. Toluene in a Mixture

Purcell and colleagues conducted a series of gas uptake studies with a binary mixture of toluene and benzene at different initial concentrations [21]. The temporal change in the gas chamber concentrations was analyzed with a rat PBPK model that has a metabolic interaction term defined in the liver compartment. Purcell and colleagues found that the non-competitive inhibitory equation provided the best simulation fit to all experimental data, suggesting that toluene was a better inhibitor of benzene metabolism than benzene was of toluene metabolism [21]. Building upon this work, Tardif and colleagues assessed the metabolic interactions of a ternary mixture of toluene, *m*-xylene, and ethylbenzene (TEX) with a rat PBPK model and gas uptake studies [22]. After determining that the competitive metabolic inhibition was the most plausible mechanism of interaction for the ternary mixture, the rat model was scaled to a human model. Based on the human model simulations and human volunteer studies, Tardif and colleagues found that the alveolar air concentrations and urinary metabolite concentrations of TEX were not significantly different between individual and combined exposures at atmospheric concentrations that were within the permissible concentrations [22].

In later studies, Haddad and colleagues developed a PBPK mixture modeling framework that allows for adding or substituting chemicals to an existing mixture model by characterizing the new binary interactions between the new chemical and pre-existing mixture components [23,24]. More specifically, Haddad and colleagues added a pre-established PBPK model for benzene (B) to the TEX mixture model [23]; and later added a pre-established PBPK model for dichloromethane (D) to their BTEX model [24]. The structure and parameter values of the existing models remain unchanged, except that the competitive inhibition of hepatic metabolism at the binary level was added to link an individual model to a mixture model. Both the BTEX and the DBTEX models were able to predict the time course of venous blood concentrations in rats following a 4-h inhalation exposure to various mixtures [23,24]. These studies demonstrated a “bottom-up” mixture modeling methodology that uses available data on binary chemical interactions to link existing PBPK models of single chemicals to predict their PK consequences in a complex mixture. When data on binary chemical interactions are not available, this modeling methodology may still be used with tools such as quantitative structure-activity relationship (QSAR) to simulate chemical interactions in a mixture. For example, Price and Krishnan recently demonstrated the use of QSAR to estimate partition coefficients, maximum rates of metabolism (V_Max_), and Michaelis constants (K_m_) based on chemical structure for 53 volatile organic compounds (VOCs) [25]. They then set the metabolic inhibition constant equal to K_m_ in a mixture PBPK model to predict the inhalation pharmacokinetics of VOCs in various mixtures. This study demonstrated the use of QSAR in PBPK modeling to provide first-cut evaluations of the kinetics of mixtures of VOCs in rats [25].

For a mixture that contains a large number of chemicals, such as gasoline, the bottom-up approach is not practical since it requires data on binary chemical interactions for all components in the mixture. Rather, a “top-down” (or lumping) approach can be used when it is not necessary to distinguish a specific chemical from the mixture (e.g., no relevant toxicity data), and when the properties of lumped chemicals can be described by a central estimate [26]. For example, Dennison and colleagues developed a mixture PBPK model that consists of five target chemicals (BTEX and n-hexane) and a lumped chemical group that represents all other chemicals in gasoline [26]. Similar to the studies described above, individual PBPK models were linked by describing the competitive inhibition of hepatic metabolism at the binary level. Using the PBPK model and the lumping approach, Dennison and colleagues were able to predict the pharmacokinetic behaviors of the five target chemicals in rats that were exposed to single chemicals and mixtures in closed-chamber studies [26].

### 3.2. Increased Tissue Dose in the Presence of Other Chemicals

#### 3.2.1. Carbon Tetrachloride (CCl_4_) and Methanol

From animal studies, the main adverse effects associated with inhaled CCl_4_ exposure are central nervous system depression and liver/kidney damage, with liver being the most sensitive target [27]. Most of the toxic effects of CCl_4_ are related to its metabolism by cytochrome P450 oxygenase, primarily CYP2E1. In addition to metabolism, Mehendale suggested that CCl_4_-mediated hepatotoxicity could be potentiated by any mechanism that destructs hepatocellular regenerative capacity [28]. In this example, Evans and Simmons tested, using gas uptake studies and a mixture PBPK model, the hypothesis that the induction of CCl_4_ metabolism is the primary mechanism involved in potentiation of CCl_4_ hepatotoxicity in rats when they were pre-treated with methanol [29]. First, the maximum metabolic rates (V_max_) of CCl_4_ in the PBPK model was estimated by fitting the model predictions with the uptake of CCl_4_ observed in the gas uptake studies under two conditions: With and without methanol pretreatment [29]. This strategy allowed enzyme induction after methanol pretreatment (assuming CYP2E1 is the only isozyme involved) to be modeled with increased V_max_ value. Their modeling results did show that V_max_ was significantly increased when rats were pre-treated with methanol, indicating a potential PK interaction between the methanol and CCl_4_.

Subsequently, Evans and Simmons examined the alteration in CCl_4_ hepatotoxicity by comparing the serum markers of alanine aminotransferase (ALT) and sorbitol dehydrogenase (SDH) in rats that were exposed to the binary mixture, and in those that were exposed to CCl_4_ alone [29]. Since overt hepatotoxicity was not observed with inhaled methanol alone [30], an increased ALT/ SDH level from the mixture exposure would suggest that potentiation of CCl_4_ hepatotoxicity are due to rats being exposed to the binary mixture. Evans and Simmons observed a dose-dependent increase in serum ALT and SDH levels when rats were exposed to both methanol and CCl_4_ [29]. This increase was significantly higher when compared to ALT and SDH levels from CCl_4_-alone exposure. In addition, it was found that given the same mixture concentration, serum ALT and SDH levels were higher at 24 h post-exposure than at 48 h post-exposure [29]. This time difference (24 *vs.* 48 h) in ALT and SDH levels suggested that not only is there a PK interaction, but also a PD interaction between the methanol and CCl_4_. In addition to metabolic induction, Evans and Simmons proposed that Kupffer cell activation might be involved in enhanced CCl_4_ hepatotoxicity with methanol co-exposure [29].

#### 3.2.2. Mirex, Phenobarbital, Chlordecone and Bromotrichloromethane (BrCCl_3_)

In this example, using PBPK modeling and gas uptake studies, Thakore and colleagues examined the effect of dietary pretreatment with Mirex, Phenobarbital, and Chlordecone on the metabolism of BrCCl_3_ [31]. The change of metabolic rate constants in a BrCCl_3_ PBPK model was examined by fitting the model predictions with the decline in the chamber concentrations of BrCCl_3_, with and without pretreatment of other chemicals. Similar to the previous example of CCl_4_, this study also evaluated the increased BrCCl_3_ hepatotoxicity following pretreatment of other chemicals. It was found that the mild enhancement of BrCCl_3_ toxicity by Mirex and Phenobarbital correlated with an increase in metabolism; but the marked potentiation seen after chlordecone pretreatment could not be attributed to the induction of BrCCl_3_ metabolism [31]. Additional experimental evidence indicated that this potentiation phenomenon is a result of chlordecone interfering with the initial tissue repair process that follows BrCCl3-induced liver injury [28]. This is another example of chemical interactions at both PK and PD levels.

## 4. Examples of Pharmacodynamic Interactions among Mixtures

### 4.1. Decreased Tissue Response in the Presence of Other Chemicals

The first example of PD interaction is a modeling exercise on identifying the interaction thresholds for chlorpyrifos and parathion mixture [32]. Chlorpyrifos and parathion both belong to the organophosphates (OP) family; they are potent pesticides that inhibit acetylcholinesterase (AChE) of many agricultural and household pests. They are found together in the environment, and humans may be exposed to these pesticides through oral, dermal and inhalation routes. Potential adverse effects following exposures include neurological, developmental, cardiac, respiratory, hepatic, hematological, metabolic, muscular, and pancreatic effects, among which neurological effects are of the most concern [33]. The mechanism of neurotoxicity for chlorpyrifos and parathion is similar—the active metabolites of both pesticides inhibit AChE resulting in prolonged stimulation of the acetylcholine receptors on the postsynaptic cells, leading to the subsequent neurotoxic effects. The competition in inhibiting AChE by these active metabolites is an interaction at the PD level.

Besides having a similar mechanism in AChE inhibition, chlorpyrifos and parathion also have similar metabolic pathways. Chlorpyrifos is rapidly desulfurated by CYP450 3A4 and 2D6 to chlorpyrifos-oxon [34,35]. Chlorpyrifos-oxon is 300 to 400 times more potent at inhibiting rat brain AChE than chlorpyrifos [36]. Parathion is desulfurated by P450 3A4, 3A5, 1A2, and 2D6 to paraoxon in liver [37,38]. Paraoxon is also a much more active inhibitor of AChE than its parent. Since the same isoenzymes P450 3A4 and 2D6 are involved in the metabolism of both chemicals to the oxon that inhibits AChE, El-Masri and colleagues used a mixture PBPK/PD model that consists of four individual sub-models (chlorpyrifos, chlorpyrifos-oxon, parathion, paraoxon) to evaluate the PK and PD interactions between chlorpyrifos and parathion [32].

El-Masri and colleagues described competitive inhibition in the two parent models, which were linked to their metabolite models in the liver compartments [32]. The predicted concentrations of the metabolites in blood were linked to a PD model for AChE kinetics where the competition for cholinesterase occurs. Partition coefficients, metabolic, and biochemical parameters in the model were obtained from the literature. Binding constants for both chlorpyrifos-oxon and paraxon to AChE were optimized to fit inhibition data found in the literature. The calibrated model was then used to determine the presence of an interaction threshold for AChE inhibition between chlorpyrifos and parathion when administered orally. In this study, the interaction threshold was determined by comparing the area under the free AChE activity curve (AUC) of the mixture exposure and the added AUCs of the chlorpyrifos-oxon only exposure and the paraoxon only exposure. As expected, a decrease of tissue response (e.g., AChE inhibition) was exhibited at high oral dose exposure to the binary mixture, and the inhibition interaction became smaller as the dose reduced [32]. A similar PBPK/PD modeling approach was used by Timchalk and Poet to evaluate the binary mixture of chlorpyrifos and diazinon, which is another OP [39]. The mixture model described the metabolic interactions using inhibition kinetics, and described B-esterase metabolism and cholinesterase inhibition as dose-additive. The model was able to simulate, in rats, the time-course of both chemicals and their metabolites in blood, as well as cholinesterase inhibition in plasma, red blood cells, and brain, following oral exposures to the mixture [39]. Similar PK and PD interactions can also be found in mixture of pesticides from different families. Carbamates, just like OP, inhibit AChE and are metabolized by cytochrome P450-mediated monooxygenases. In this next example, the PK and PD interactions between carbaryl (a carbamate pesticide) and chlorpyrifos are discussed. Carbaryl and chlorpyrifos are both widely used pesticides for which individual PBPK models have been developed [40,41]. An important distinction between these two pesticides is that while the interaction of chlorpyrifos-oxon with AChE is essentially irreversible, the interaction of carbaryl (which is the active AChE inhibitor) with AChE is rapidly reversible. To study the binary mixture of carbaryl and chlorpyrifos, the PBPK models for carbaryl and chlorpyrifos were linked through descriptions of competitive inhibition at sites of metabolism and at AChE [42]. The linked mixture model predicted a complex time course for AChE that reflects the combination of the rapid but reversible binding of carbaryl to AChE together with the slower, irreversible binding of chlorpyrifos-oxon to AChE.

### 4.2. Increased Tissue Response in the Presence of Other Chemicals

Potentiation of CCl_4_ hepatoxicity is used again to discuss the role of PD interactions among chemicals. To explain the different potentiated CCl_4_ hepatotoxicity when co-exposed to Kepone, (chlordecone). Mehendale proposed a “two-stage model of toxicity” concept. In the first stage, cellular and/or tissue injury inflicted by toxic chemicals evokes homeostatic mechanisms, such as cellular proliferation and tissue repair, to restore the original tissue structure [43]. With no additional toxic assaults, complete and prompt recovery is expected from the toxicant-induced injury. Blocking or pertubing the homeostatic mechanism with additional exposures, however, would lead to the second stage of toxicity where progression of extensive injury occurs. This concept could explain the marked amplification of CCl_4_ hepatotoxicity and lethality following pre-exposure to a non-toxic level of Kepone [44,45] or other halomethanes [28,43].

Lockard and colleagues exposed rats to 0.1 ml/kg CCl_4_ via a single intraperitoneal injection, and they observed limited hepatocellular necrosis accompanied by ballooned cells and steatosis [46,47]. Within 6 h after exposure, liver tissues responded to the toxicity by stimulating hepatocellular regeneration and tissue repair [46,47]. The repair mechanism continued after the hepatocellular necrosis advanced to a more progressive phase between 6 and 12 h following exposure. In addition to restoring the hepatolobular structure by replacing dead cells, the newly generated cells also demonstrated resistance to the existed toxicity [48]. With these more resistant new cells, the regenerated liver was able to endure a greater assault in the progressive phase and fully recover later [43].

But, if animals which were administered the same dose of CCl_4_ were pretreated with 10 ppm Kepone, the liver injury observed in these animals became much higher compared to that observed in animals exposed to either chemical alone [46,47]. Kodavanti and colleagues suggested that the pre-treatment of Kepone suppressed the initial hepatocellular regeneration and thus resulted in two consequences: (1) The hepatolobular structure cannot be restored; and (2) Liver injury at the progressive phase accelerated in the absence of those newly divided, relatively resistant cells [49]. This hypothesis was further investigated in a Kepone/CCl_4_ mixture study with postnatal rats [50]. While rat pups at 2, 5, 20, and 35 days of age were completely resilient to Kepone potentiation of CCl_4_ toxicity, young rats by 60 days of age were as sensitive as adult rats. The hepatic microsomal cytochrome P450 levels in the 35-, 45-, and 60-day-old rats exposed to Kepone were no different from each other, suggesting that PK interaction between Kepone and CCl_4_ cannot explain the observed discrepancy in potentiation of CCl_4_ toxicity between 35- and 60-day-old rats. It is more likely that the resiliency of younger rats to Kepone-potentiated CCl_4_ toxicity was related to the active hepatocellular regeneration during the early development stage [50].

In a later study, El-Masri and colleagues constructed a PBPK/PD model that includes the following three effects of Kepone on CCl_4_ hepatotoxicity: (1) Inhibition of mitosis; (2) Reduction of the repair mechanism of hepatocellular injury; and (3) Suppression of phagocytosis [51]. The values for PD parameters in the model were estimated by fitting the model simulations to fractions of injured, pyknotic, and mitotic cells from rats exposed to CCl_4_ with and without Kepone [47]. The calibrated model was then used to predict the LD_50_ (the dose required to kill half of a tested population) for CCl_4_ toxicity. The model predictions were consistent with the observed mortality, showing a ∼60-fold amplification of CCl_4_ lethality in the presence of Kepone.

In another study, animals were co-exposed to CCl_4_ and Phenobarbital [48]. Enhanced CCl_4_ hepatotoxicity was observed, but no significantly increase lethality was found as in the case of Kepone/CCl_4_ co-exposure. The enhanced hepatotoxicity may be caused by the induction of cytochrome P450 CYP2E1, which leads to increasing CCl_4_ bioactivation. But, unlike Kepone, Phenobarbital did not compromise the capability of hepatocellular regeneration. Thus, the potentiated CCl_4_ hepatotoxicity was able to be reversed, though being delayed, by stimulated hepatocellular regeneration and tissue repair mechanism [43]. Both Kepone and Phenobarbital potentiate CCl_4_ hepatotoxicity, but the ultimate outcome (lethality or reversible *vs.* irreversible toxicity) is determined by the interaction mechanisms.

## 5. Applications in Assessing Human Health Risks

Most studies reviewed in Sections 3 and 4 were conducted in animals. Only a few studies used computational models to examine the effects of PK interactions among chemicals in a mixture for human health risk assessment. These studies were all conducted in occupational settings, in which potential exposure concentrations may be high enough for interactions to occur. Some examples are presented below.

Occupational health and safety professionals use a “unity calculation” to evaluate whether over-exposure occurs when workers are exposed to mixtures of chemicals that share a common mechanism of toxicity [52]. The unity calculation first converts the exposure concentration of each chemical in the mixture to a fraction of the corresponding reference occupational exposure limit (OEL) (*i.e.*, exposure concentration divided by the OEL). If the sum of these fractions (denoted as EM) exceeds unity (1.0), then the exposures should be reduced. This method assumes dose additivity, and it does not take into account PK or PD interactions. In a study conducted by Dennison and colleagues, the effect of PK interactions was factored in by using a human PBPK model for a mixture of toluene, ethylbeneze, and xylene to predict venous blood concentrations for a variety of exposure concentrations, as well as for different activity levels [53]. The predicted blood concentrations, instead of inhaled concentrations, were then used to derive an ‘internal dose-based EM’, which is the sum of each chemical’s blood concentration at the exposure concentration divided by that chemical’s blood concentration at the OEL. They found that the traditional unity calculation that omits PK interactions can lead to significant over-exposures [53]. For example, in an exposure scenario based on exposure to one-third of the permissible exposure limit (PEL) for each chemical, the traditional unity calculation would result in an EM of 1.0 (= 1/3 + 1/3 + 1/3). However, the EM calculated based on blood concentrations was 2.9, suggesting that the cumulative blood concentrations of the three chemicals would be three times the concentration allowed by the PELs [XX]. Dennison and colleagues also found that workers with higher activity levels may experience significantly higher absorbed doses [53].

In another study, Jang and colleagues used PBPK modeling to investigate the interaction effects of a binary exposure to ethylbenzene and xylene for male workers who had been exposed to this mixture during painting and solvent mixing [54]. The PBPK model-predicted urinary excretion of methylhippuric acid (a metabolite of xylene) were 4.94 and 1.55 g/g creatinine when workers were exposed to 100 ppm of pure xylene and a mixture of 100 ppm of xylene and 25 ppm of ethylbenzene, respectively. The predicted result was consistent with that (1.96 g/g creatinine) calculated using the linear regression equation obtained from measured xylene and ethylbenzene concentrations in air and measured methylhippuric acid in urine [54]. Jang and colleagues concluded that PK interactions due to mixture exposures can significantly complicate the interpretation of biomonitoring data, and thus, PK interactions should be considered when developing biological limit values (BLVs) for mixture exposures [54].

As reviewed in Section 3.1.1, Dobrev and colleagues used a rat PBPK model to investigate the reduction of TCE metabolism caused by co-exposure to PERC and MC [14]. Dobrev and colleagues later scaled up the rat model to a human model for evaluating the impact of metabolic interactions in humans and its implications for risk assessment [55]. They found that inhibition of TCE metabolism in the presence of PERC and MC resulted in decreased formation of oxidative metabolites, but increased the formation of conjugative metabolites, S-1,2-dichlorovinylcysteine (S-1,2-DCVC) and S-2,2-DCVC. Both oxidative and conjugative metabolites of TCE have been associated with adverse effects in animals [56]. This study clearly demonstrated that human health risk associated with exposures to chemical mixtures is complexly related to the mechanism of interactions and the identity of the toxic moiety (e.g., parent or metabolite) [55].

A similar conclusion was reached in a study that used a PBPK model for DBTEX to conduct both non-cancer and cancer risk assessments [57]. In this study, Haddad and colleagues scaled up a rat DBTEX model (reviewed in Section 3.1.2; [24]) to a human model to account for the effects of PK interactions on tissue dose metrics [57]. For the non-cancer risk assessment, the PBPK model predicted AUC of D, B, T, E, and X in the richly perfused tissue compartment (representing brain) were used as dose metrics for CNS effects; and the PBPK model predicted AUC of carboxyhemoglobin in blood was used as a dose metric for hypoxia [57]. These dose metrics were used to calculate an ‘interaction-based’ hazard index (HI) for comparison with the conventional HI based on dose addition. Haddad and colleagues showed that, at high exposure concentrations, the interaction-based estimates of HI were higher for CNS effects, but lower for hypoxia [57]. For cancer risk assessment, Haddad and colleagues used the PBPK model-predicted amount of GSH conjugates as the dose surrogate for D and predicted the amount metabolized as the dose surrogate for B. Their analyses showed that in the presence of competitive inhibitors of P450 metabolism (B/D, T, E, X), cancer risk attributed to D exposure increased; but cancer risk attributed to B exposure decreased [57].

Human PBPK models presented in the above examples were all scaled up from existing animal models. Human PBPK models can also be developed using *in vitro* data. For example, Haddad and colleagues developed a “physiologically based” liver model based on *in vitro* data to describe the inhibition, metabolism, transport, and partitioning of *R*-bufuralol, bunitrolol, and debrisoquine in the liver [XX]. The model was used to simulate the perfusate kinetics of each drug in an isolated perfused rat liver for the single and paired drugs. In Comparison with empirical models, the physiologically based liver model, overall, performed the best [58]. Although this work was on drug-drug interactions, the approach for the development and subsequent application of a PBPK model for investigating chemical interactions is also applicable to environmental chemicals. The use of PBPK modeling in predicting drug-drug interactions can be found elsewhere (e.g., [59–61]).

The above examples represent experience with applying human PBPK models for chemical mixtures in cumulative risk assessment for predicting conditions under which PK interactions are likely to alter the assumption of dose additivity. Human PD models, however, are yet to be utilized with PBPK models to assess responses associated with exposures to chemical mixtures that have common modes of action (e.g., cholinesterase inhibited by chlorpyrifos and carbaryl [Section 4.1]). Furthermore, the emerging discipline systems biology may assist in understanding how mixtures of chemicals affect a common physiological endpoint (e.g., interference with endocrine) by either similar or diverse modes of action in the body [6]. Systems biology studies biological systems by globally monitoring the gene, protein, and pathway responses to perturbations, integrating these data and formulating mathematical models to describe the structure of the system and its response dynamics [62]. Since the mode of action is related to the perturbation of biological systems, systems biology can help ascertain modes of actions that involve different pathway targets within cells, tissues, or organs [6]. Using PBPK/PD models of these perturbations allows us to expand the current cumulative risk assessment paradigm to focus on the biology of responses more than on the kinetics of the chemicals [6].

## 6. Conclusions

Environmental exposure to multiple chemicals simultaneously or sequentially is the rule rather than an exception. When conducting cumulative risk assessment for chemicals in a mixture, the PK and PD interactions among chemicals need to be characterized since these interactions may cause alterations in the toxicity predicted based on the summation of the effects of each chemical. PK interactions occur when one chemical alters the absorption, distribution, metabolism, and/or excretion of other chemical(s). PD interactions occur when one chemical alters the tissue response of other chemical(s). The knowledge on PK and/or PD interactions can be integrated in a quantitative manner with a PBPK/PD model. A PBPK model can be used for dose, route and interspecies extrapolations of the target tissue concentration of the toxic moieties. A PD model can be used for describing mechanisms of action and tissue responses. An integrated PBPK/PD model for studying chemical interactions at both the level of PK (e.g., metabolic interactions) and PD (e.g., receptor interactions) is imperative to achieve the ultimate goal of assessing the health risks associated with human exposure to complex chemical mixtures. More than a science of observation, toxicology should be a science of observation and analysis.
